# Supplementary material for: Combining RNA-seq data and homology-based gene prediction for plants, animals and fungi
Source: BMC Bioinformatics. 2018 May 30;19:189. doi: 10.1186/s12859-018-2203-5 (PMC5975413; doi:10.1186/s12859-018-2203-5)
Supplement: Supplementary file 1 — Supplementary Tables and Figures. Table S1: Data used for the BRAKER1 benchmark. Table S2: Data for Wormbase study. Table S3: Data for barley annotation. Table S4: F1-measure on the BRAKER1 test sets. Table S5: CodingQuarry and GeMoMa results for S. pombe using the original read mappings from [10, 11]. Table S6: CodingQuarry and GeMoMa results for S. cerevisiae. Table S7: GeMoMa runtime. Table S8: GeMoMa performance for D.melanogaster. (PDF 147 kb) [file 12859_2018_2203_MOESM1_ESM.pdf]

Target organism	Genome	Reference organism	Genome	Annotation
A. thaliana (TAIR10)	FastA	*A. lyrata* (Ensembl 31)	FastA	GFF
C. elegans (WS240)	FastA	*C. briggsae* (WS257)	FastA	GFF
D. melanogaster (r6.07)	FastA	*D. simulans* (r2.02)	FastA	GFF
		D. sechellia (r1.3)	FastA	GFF
		D. yakuba (r1.05)	FastA	GFF
		D. erecta (r1.05)	FastA	GFF
		D. ananassae (r1.05)	FastA	GFF
		D. persimilis (r1.05)	FastA	GFF
		D. willistoni (r1.05)	FastA	GFF
		D. grimshawi (r1.05)	FastA	GFF
S. pombe (ASM294v2.23)	FastA	*S. octosporus* (Ensembl 31)	FastA	GFF

Additional File 1 – Tab. S 1: Data used for the BRAKER1 benchmark. We used the same version of the target species as in the BRAKER1 publication enabling a direct comparison. The table contains links to the files.

Target organism	Genome	Annotation	RNA-seq
C. briggsae	FastA	GFF	SRX392707, SRX392718, SRX392710, SRX392711, SRX392708, SRX392716, SRX089119, SRX103655, SRX089120, SRX100088, SRX089069, SRX100089, SRX103667, SRX100087, SRX100086, SRX089070, SRX127749, SRX127748
C. remanei	FastA	GFF	SRX052082, SRX052083, SRX101879, SRX101880, SRX101881, SRX101882, SRX101883, SRX101884, SRX101885, SRX101886, SRX101887, SRX101888, SRX101889, SRX101890, SRX101891, SRX101892, SRX101893, SRX101894, SRX101895, SRX191971, SRX191972, SRX191973, SRX191974, SRX191975, SRX191976, SRX311798, SRX311799, SRX311800, SRX311801, SRX311802, SRX311803, SRX510986, SRX510987, SRX510988, SRX510989, SRX510990, SRX510991, SRX510992, SRX510993, SRX510994, SRX510995, SRX510996, SRX510997, SRX510998, SRX510999, SRX511000, SRX511001, SRX511002, SRX511003, SRX511004, SRX511005, SRX511006, SRX511007, SRX511008, SRX511009, SRX511010, SRX511011, SRX511012, SRX511013, SRX511014, SRX511015, SRX511016, SRX511017, SRX511018, SRX511019, SRX511020, SRX511021, SRX747479, SRX747480, SRX747481, SRX747482, SRX747483, SRX747484, SRX747485, SRX747486, SRX747487, SRX747488, SRX747489, SRX747490
C. japonica	FastA	GFF	SRX100090, SRX100091, SRX100092, SRX100093, SRX100094, SRX100095, SRX191959, SRX191960, SRX191961, SRX191962, SRX191963 ,SRX191964
C. brenneri	FastA	GFF	SRX100769, SRX100770, SRX100771, SRX100772, SRX100773, SRX100774, SRX100775, SRX100776, SRX100777, SRX100778, SRX100779, SRX100780, SRX103654, SRX103657, SRX191965, SRX191966, SRX191967, SRX191968, SRX191969, SRX191970, SRX311810, SRX311811, SRX311812, SRX311813, SRX311814, SRX311815, SRX565023, SRX565024, SRX565025, SRX565026, SRX565027, SRX565028, SRX565029, SRX565030, SRX565031, SRX565032,
C. elegans	FastA	GFF	

Additional File 1 – Tab. S 2: Data for Wormbase study. We used version WS257 for all organisms. The table contains links to the files.

Target organism	Version
A. thaliana	TAIR10
B. distachyon	3.1
O. sativa	7.0
S. italica	2.2

Additional File 1 – Tab. S 3: Data for barley annotation. Genome and annotation per reference organism were downloaded from Phytozome.

	MAKER2$^+$ (exonerate)	GeMoMa$^+$ without RNA-seq	GeMoMa$^+$ with RNA-seq	RNAseq-Cufflinks	RNAseq-StringTie	BRAKER1*	MAKER2*	CodingQuarry*	MAKER2$^+$ (exonerate, Trinity, Augustus)	MAKER2$^+$ (GeMoMa, Trinity, Augustus)
Arabidopsis thaliana (ref. *A. lyrata*)										
Gene	0.46	0.63	**0.69**	0.36	0.45	0.58	0.52	NA	0.61	0.62
Transcript	0.42	0.58	**0.61**	0.30	0.40	0.53	0.48	NA	0.56	0.57
Exon	0.75	0.83	0.84	0.68	0.72	0.81	0.76	NA	**0.85**	**0.85**
Caenorhabditis elegans (ref. *C. briggsae*)										
Gene	0.31	0.44	**0.55**	0.23	0.28	**0.55**	0.35	NA	0.45	0.51
Transcript	0.27	0.38	0.47	0.19	0.24	**0.48**	0.31	NA	0.39	0.44
Exon	0.63	0.72	0.76	0.65	0.69	**0.83**	0.66	NA	0.77	0.81
Drosophila melanogaster (ref. *D. simulans*)										
Gene	0.67	0.80	**0.85**	0.63	0.63	0.62	0.50	NA	0.65	0.68
Transcript	0.54	0.64	**0.72**	0.54	0.56	0.51	0.42	NA	0.53	0.55
Exon	0.78	0.83	**0.86**	0.76	0.76	0.78	0.67	NA	0.81	0.82
Schizosaccharomyces pombe (ref. *S. octosporus*)										
Gene	0.54	0.80	**0.83**	0.80	0.77	0.79	0.53	0.76	0.79	0.81
Transcript	0.54	0.80	**0.83**	0.74	0.68	0.77	0.53	0.76	0.79	0.81
Exon	0.64	0.85	**0.87**	0.82	0.80	0.83	0.59	0.81	0.85	**0.87**

Additional File 1 – Tab. S 4: F1-measure on the BRAKER1 test sets. The target species are given in multi-column rows. The same reference species, which is given in brackets, is used for all tools using homology-based gene prediction indicated by plus. The asteriks indicates that the performance of BRAKER1, MAKER2 and CodingQuarray is given as reported in Hoff *et al.* (2016). The highest value per line is depicted in bold-face.

	CodingQuarry			GeMoMa	
Measure	published	Testa *et al.*	Hoff *et al.*	Testa *et al.*	Hoff *et al.*
Gene Sn	87.5%	87.37%	78.63%	79.19%	79.00%
Gene Sp	83.0%	81.98%	71.20%	88.16%	87.92%
Transcript Sn	-	87.35%	78.61%	79.18%	78.98%
Transcript Sp	-	81.98%	71.20%	87.76%	87.48%
Exon Sn	90.3%	90.23%	80.51%	82.94%	82.87%
Exon Sp	89.4%	88.70%	80.72%	92.06%	91.83%
Nucleotide Sn	98.6%	99.20%	98.77%	95.26%	95.25%
Nucleotide Sp	98.9%	99.31%	98.97%	99.77%	99.77%

Additional File 1 – Tab. S 5: CodingQuarry and GeMoMa results for *S. pombe* using the original read mappings from Testa *et al.* (2015) and Hoff *et al.* (2016), respectively. We find that CodingQuarry is sensitive to different parameter settings for read mapping, while the performance of GeMoMa is largely stable. While GeMoMa performs better than CodingQuarry on all levels except the nucleotide level using the mapping of Hoff *et al.* (2016), we find mixed results using the mappings of Testa *et al.* (2015), where CodingQuarry obtains higher sensitivities whereas the predictions of GeMoMa yield higher specificities.

Measure	CodingQuarry (published)	CodingQuarray (re-computed)	GeMoMa (*S. eubayamus*)
Gene Sn	76.6%	77.13%	75.30%
Gene Sp	88.3%	86.70%	92.58%
Transcript Sn	-	77.13%	75.30%
Transcript Sp	-	86.70%	92.58%
Exon Sn	76.1%	77.13%	73.79%
Exon Sp	87.2%	85.63%	91.47%
Nucleotide Sn	97.2%	99.39%	97.80%
Nucleotide Sp	99.5%	99.24%	99.65%

Additional File 1 – Tab. S 6: CodingQuarry and GeMoMa results for *S. cerevisiae*. We ran GeMoMa for the prediction of protein-coding gene models in *S. cerevisiae* using the *S. eubayamus* (`ftp://ftp.ncbi.nlm.nih.gov/genomes/all/GCF/001/298/625/GCF_001298625.1_SEUB3.0/`) as reference species and the original BAM files of Testa *et al.* (2015). CodingQuarry and GeMoMa perform comparable on *S. cerevisiae*, where CodingQuarry is slightly more sensitive and GeMoMa is slightly more specific.

#transcripts	100		500		1000	
species	tblastn	GeMoMa	tblastn	GeMoMa	tblastn	GeMoMa
A. thaliana	76	28	476	65	882	110
C. elegans	85	42	387	122	759	914
D. melanogaster	253	203	1157	532	2159	852
S. pombe	19	19	78	53	158	99

Additional File 1 – Tab. S 7: GeMoMa runtime. Runtime analysis for a fixed number of transcripts using different target species. All runtimes are given in seconds and depend on the genome size. In most cases, tblastn dominates the runtime. Runtime was measured using a single core of Intel® Xeon® E5-4657L v2 2.40GHz.

	D. simulans	*D. sechellia*	*D. yakuba*	*D. erecta*	*D. ananassae*	*D. persimilis*	*D. willistoni*	*D. grimshawi*
Gene Sn	83.08%	69.09%	80.67%	80.62%	73.35%	57.93%	62.39%	64.44%
Gene Sp	87.09%	65.53%	86.86%	88.40%	83.75%	65.03%	75.89%	79.56%
Transcript Sn	64.99%	47.94%	62.57%	62.49%	56.53%	40.50%	46.67%	50.22%
Transcript Sp	81.19%	63.95%	80.77%	83.98%	78.19%	62.39%	72.97%	74.20%
Exon Sn	79.96%	70.77%	78.24%	78.17%	73.81%	62.42%	66.68%	70.25%
Exon Sp	93.31%	84.42%	93.11%	94.61%	92.40%	85.60%	90.41%	91.49%
Nucleotide Sn	91.47%	88.56%	90.30%	90.91%	88.24%	81.57%	84.27%	85.91%
Nucleotide Sp	99.16%	97.20%	98.97%	99.30%	99.18%	97.77%	99.21%	99.36%

	1 out of 8	2 out of 8	3 out of 8	4 out of 8	5 out of 8	6 out of 8	7 out of 8	8 out of 8
Gene Sn	87.63%	85.24%	81.89%	76.97%	70.69%	61.79%	49.52%	30.93%
Gene Sp	74.17%	92.90%	94.40%	95.16%	96.15%	96.86%	97.43%	97.61%
Transcript Sn	71.65%	67.89%	64.09%	58.99%	52.94%	45.39%	35.89%	22.31%
Transcript Sp	56.89%	82.69%	88.65%	91.82%	94.14%	95.49%	96.37%	97.02%
Exon Sn	88.02%	80.95%	76.43%	69.84%	62.11%	52.02%	38.92%	22.48%
Exon Sp	79.45%	95.07%	96.96%	97.80%	98.41%	98.68%	98.88%	98.98%
Nucleotide Sn	98.26%	91.60%	87.78%	82.31%	75.40%	65.86%	51.38%	31.02%
Nucleotide Sp	95.99%	99.44%	99.70%	99.81%	99.85%	99.86%	99.88%	99.85%

Additional File 1 – Tab. S 8: GeMoMa performance for *D.melanogaster* using different reference species and different level of conservation (GAF parameter *evidence percentage filter*).

References

Hoff, K. J., Lange, S., Lomsadze, A., Borodovsky, M., and Stanke, M. (2016). BRAKER1: Unsupervised RNA-Seq-Based Genome Annotation with GeneMark-ET and AUGUSTUS. *Bioinformatics*, **32**(5), 767.

Testa, A. C., Hane, J. K., Ellwood, S. R., and Oliver, R. P. (2015). CodingQuarry: highly accurate hidden Markov model gene prediction in fungal genomes using RNA-seq transcripts. *BMC Genomics*, **16**(1), 170.
